# Functional lifting assessment for determining impairment in low back pain patients compared with asymptomatic controls

**DOI:** 10.3389/fspor.2026.1843769

**Published:** 2026-07-15

**Authors:** L. Risch, T. Engel, J. Stoll, A. Hanisch, M. Cassel, F. Mayer

**Affiliations:** Department of Sports Medicine, University Outpatient Clinic, University of Potsdam, Potsdam, Germany

**Keywords:** assessment, back pain, lifting, reliability, spine

## Abstract

**Background:**

People with low back pain (LBP) commonly report functional impairment in activities of daily living, such as bending and lifting. A standardized, performance-based lifting assessment that can be applied in a clinical setting may provide a quantifiable measure for this impairment.

**Objective:**

This study investigates the usability and reliability of a lifting assessment for detecting functional impairment in individuals with LBP compared with asymptomatic controls (CTRLs).

**Methods:**

Forty-eight LBP patients [29 women/19 men, age 39 ± 11 years, height 175 ± 9 cm, weight 77 ± 16 kg, pain intensity ≥3 on the numerical rating scale (NRS; 0–10)] and 37 age- and sex-matched controls (27 women/10 men, age 39 ± 12 years, height 172 ± 10 cm, weight 73 ± 15 kg) performed four box-lifting tasks: squat and stoop lifting, each without (0%) and with additional weight (20% body weight) in a standardized sequence (squat 0%, stoop 0%, squat 20%, stoop 20%). Each lifting task was performed twice consecutively (T1 and T2). Time to task completion (TTC, s) was defined as the main outcome measure. Descriptive statistics are presented as mean ± SD. Group comparisons were analyzed pairwise and using two-way repeated-measures ANOVA with Bonferroni correction (mean of T1 and T2; *α*=0.0125). Test–retest reliability (T1 vs. T2) was assessed using the ICC, TRV%, and Bland–Altman analysis. Test performance and cut-off values were analyzed using receiver operating characteristics (ROC) curve, including the area under the curve (AUC) and Youden's index.

**Results:**

LBP patients revealed higher TTC values across all lifting conditions (4.0 ± 0.9 to 5.0 ± 1.8 s) compared with asymptomatic controls (difference of 2.8 ± 0.4 to 3.2 ± 0.4s; *p* < 0.001; interaction effect *p* = 0.005). Reliability analysis revealed ICC value ranging from 0.85 to 0.95 s, TRV values from 6% to 8%, and bias values from 0.05 to 0.34 s. ROC analysis showed an AUC ranging from 0.915 to 0.923 and threshold values from 3.5 to 3.7s.

**Discussion:**

TTC of a simple lifting task is a reliable measure that effectively differentiates people with functional limitations due to LBP from their asymptomatic counterparts, who complete the tasks more quickly and with less variability in completion time. Nevertheless, the responsiveness of this assessment remains to be clarified before its applicability in a clinical setting can be fully determined.

## Introduction

Low back pain (LBP) is a frequent symptom that affects people worldwide and across all age and income groups (1). It is the leading cause of years lived with disability and imposes a high socioeconomic burden that is likely to increase in the future (2). The highest prevalence of disability is found among the working population (1), resulting in associated costs due to production loss and absence from work (3, 4).

LBP patients commonly present with functional impairment and altered movement patterns during daily activities resulting from nociception and anticipation or fear of pain (5, 6). Several studies have investigated alterations in bending and lifting tasks, as these activities are often considered provocative for the acute onset of LBP (7, 8). Although physiotherapists and health care practitioners commonly advise specific lifting techniques, preferring squat (straight trunk and knees bended) over stoop (trunk flexed and knees less bended/straight) lifting to avoid lumbar flexion (4, 9, 10), there is no credible *in vivo* evidence that lumbar flexion during lifting is a risk factor for the onset of low back pain (10, 11) and that squat lifting has a preventive effect (4, 12, 13) or results in reduced spinal loading (14). Motion analysis has revealed that LBP patients generally lift more slowly and with greater stiffness and perform a deeper knee bend compared with pain-free controls (4). Slower movement velocity is associated with lower spinal loads. In addition, it has been suggested that movement velocity even has a higher impact on spinal loading than the applied lifting technique (14). Furthermore, longitudinal cohort and case studies have shown that shifting from squat-like to stoop-like lifting techniques, as well as an increase in trunk movement (angular) velocity during lifting (15) and during bending (16), is associated with a decrease in pain and disability. Therefore, lifting performance represents a relevant assessment for measuring the degree of functional impairment in LBP patients.

In clinical settings, functional impairment in LBP patients is commonly assessed using established functional mobility tests, such as the “timed up and go” and “chair rise” tests, which were adopted from geriatric and neurological target populations in the context of fall prevention (17–19). The applicability and validity of these tests in frequently affected LBP patients in the working population may be limited, given that the original target population consists of elderly or disabled people. Therefore, adding an inexpensive, easy-to-administer, reliable, and valid functional lifting test focusing on unloaded and loaded everyday lifting movements may help determine pain-related functional impairments and monitor improvement in the condition over time. Previous studies have applied lifting tasks consisting of repetitive, progressive lifting within a predefined time frame with absolute or categorical performance ratings and have yielded moderate to good reliability and construct validity but limited or questionable responsiveness (20). As LBP patients commonly move more slowly and more carefully, a simple timed lifting task, similar to other clinical tests (21), may be an effective measure. This study aimed to investigate whether time to completion (TTC) of a standardized unloaded and loaded squat and stoop lifting test provides a reliable assessment and whether it discriminates between LBP patients and asymptomatic controls, hypothesizing that LBP patients require a longer time for task completion.

## Methods

### Participants and study design

In this cross-sectional study, 56 LBP patients with a current back pain rating of at least 3 on a numerical rating scale (NRS 0–10, at rest or during activity) and asymptomatic age- and sex-matched controls with no history of LBP were recruited from the university outpatient clinic and included after providing written informed consent. After exclusion of data from eight LBP patients because of missing values, 48 LBP patients were compared with 37 asymptomatic controls. The study was approved by the local ethics committee.

All participants were clinically examined by a sports orthopedic physician to ensure appropriate inclusion and safe participation. Following the clinical examination, anthropometric data, training-related data, and current pain rating on an NRS during rest and activity were obtained.

### Functional lifting tasks

**Figure 1:**
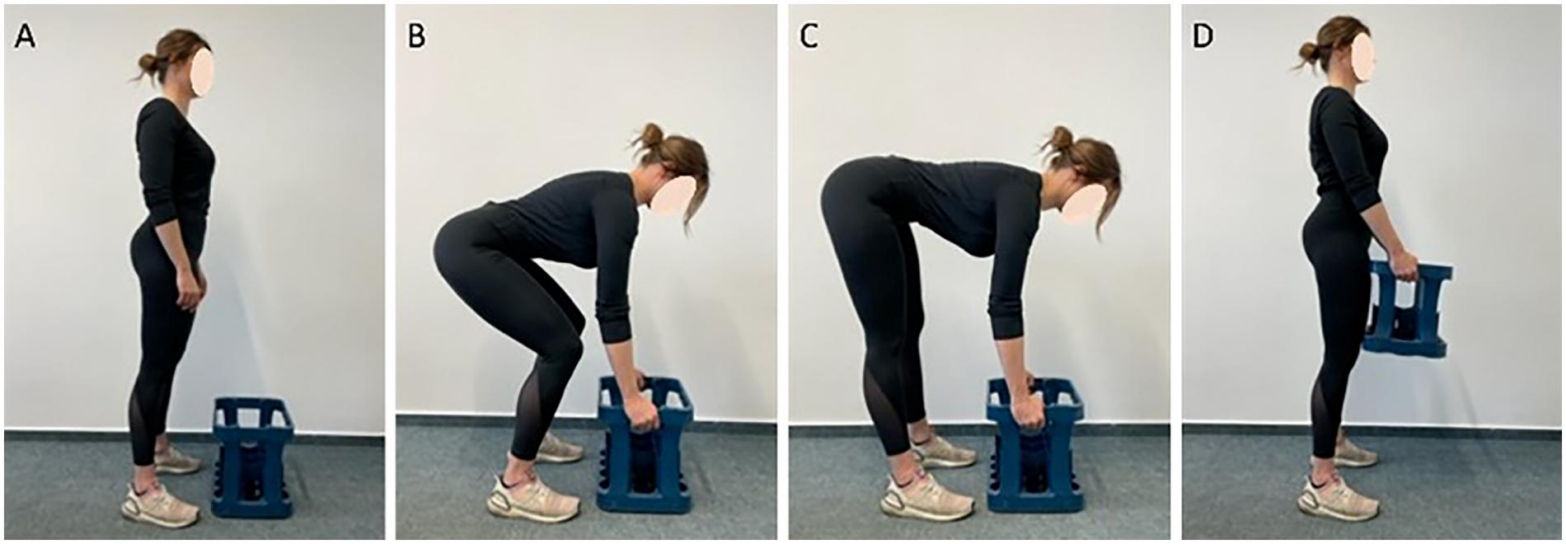
Functional lifting test: **(A)** start/end position, **(B)** squat lifting, **(C)** stoop lifting, **(D)** middle position; time to completion is in seconds.

All patients received a demonstration and verbal instructions from the examiner on how to perform the lifting test procedure. Every lifting task started from an upright position (Figure 1A), with markers for toe position (behind the box) and box placement, followed by lifting the box from the ground in squat (Figure 1B) or stoop (Figure 1C) manor to an upright position with extended hips (Figure 1D), returning the box to the ground (in front of them, no specific location), and ending in the upright position. Time from the start to the end position (Figure 1A) was measured manually with a stopwatch (in seconds). The examiner started the timer on the verbal command “go” and stopped it as soon as the participant returned to the fully upright starting position. The lifting conditions were performed in the following order: squat lifting without additional weight (Squat0%), stoop lifting without additional weight (Stoop0%), squat lifting with additional weight (20% of body weight, Squat20%), and stoop lifting with additional weight (20% of body weight, Stoop20%). Additional weight was added by placing free weights in the box. All participants performed one practice trial (only for each unloaded condition), followed by two subsequent test trials (T1 and T2) for each of the four conditions. Every trial was separated by a standardized rest period of 15 s.

### Data analysis

To analyze test–retest reliability, the TTC (s) for T1 and T2 was compared. For comparison between the LBP and control groups, the mean time of the two repetitions (T1 and T2) was used to provide a more stable assessment.

### Statistical analysis

Descriptive statistics for TTC are presented as mean ± SD with 95% confidence interval (CIs) and median with interquartile range (IQR: 25th and 75th percentiles). Group differences across the four functional lifting conditions were analyzed using parametric or non-parametric pairwise tests after checking for normality. Interaction effects were analyzed using two-way repeated-measures ANOVA for groups (2) and conditions (4). The correlation between the degree of highest reported pain intensity and TTC was tested with Pearson correlation. Reliability of the test–retest measurements (T1, T2) was assessed using the intraclass correlation coefficient (ICC 2.1), test–retest variability (TRV, %; (*|x_i_−y_i_|*/ ½(*x_i_* *+* *y_i_*))×100, where *x_i_* is the time of completion of T1 and *y_i_* is the time of completion of T2 for subject *i*), standard error of measurement (SEM), smallest detectable change (SDC, SEM*1.96), and Bland–Altman analysis (bias ± LoA). Receiver operating characteristic (ROC) analysis, including area under the curve (AUC, 95%CI) and Youden's *J* statistics (*J* = sensitivity + specificity−1) (22), was used to determine test performance and the cut-off value for differentiating between controls and LBP patients. The alpha level was *α*=0.05, and Bonferroni correction was used for multiple pairwise testing (*α*=0.0125).

## Results

### Participants

Participant characteristics are presented in Table 1. There were no significant differences in anthropometrics between the LBP and control groups (Table 1). Of the 56 LBP patients, 48 (85%) completed all four testing conditions. Eight LBP patients (15%) refused to participate in at least one task because of fear of pain (*n* = 2 declined all lifting tasks, *n* = 1 declined both squat20% and stoop20%, and *n* = 5 declined stoop20%). These participants were excluded from further analysis. In the control group, all 37 participants completed all four testing conditions.

**Table 1:** Group characteristics presented as number (*n*), mean ± SD, or median (IQR).

Groups	Sex	Age (years)	Height (cm)	Weight (kg)	Pain NRS (rest/activity)
LBP patients	29f/19m	39 ± 11	175.3 ± 9.0^a^	77.4 ± 16.6	3 (3,4)/5 (4,6)
Controls	27f/10m	39 ± 12	172.2 ± 10.3	72.8 ± 15.0	0/0
*p*-Value		0.915	0.180	0.194	

a Non-parametric testing.

### Discrimination LBP and control groups

**Figure 2:**
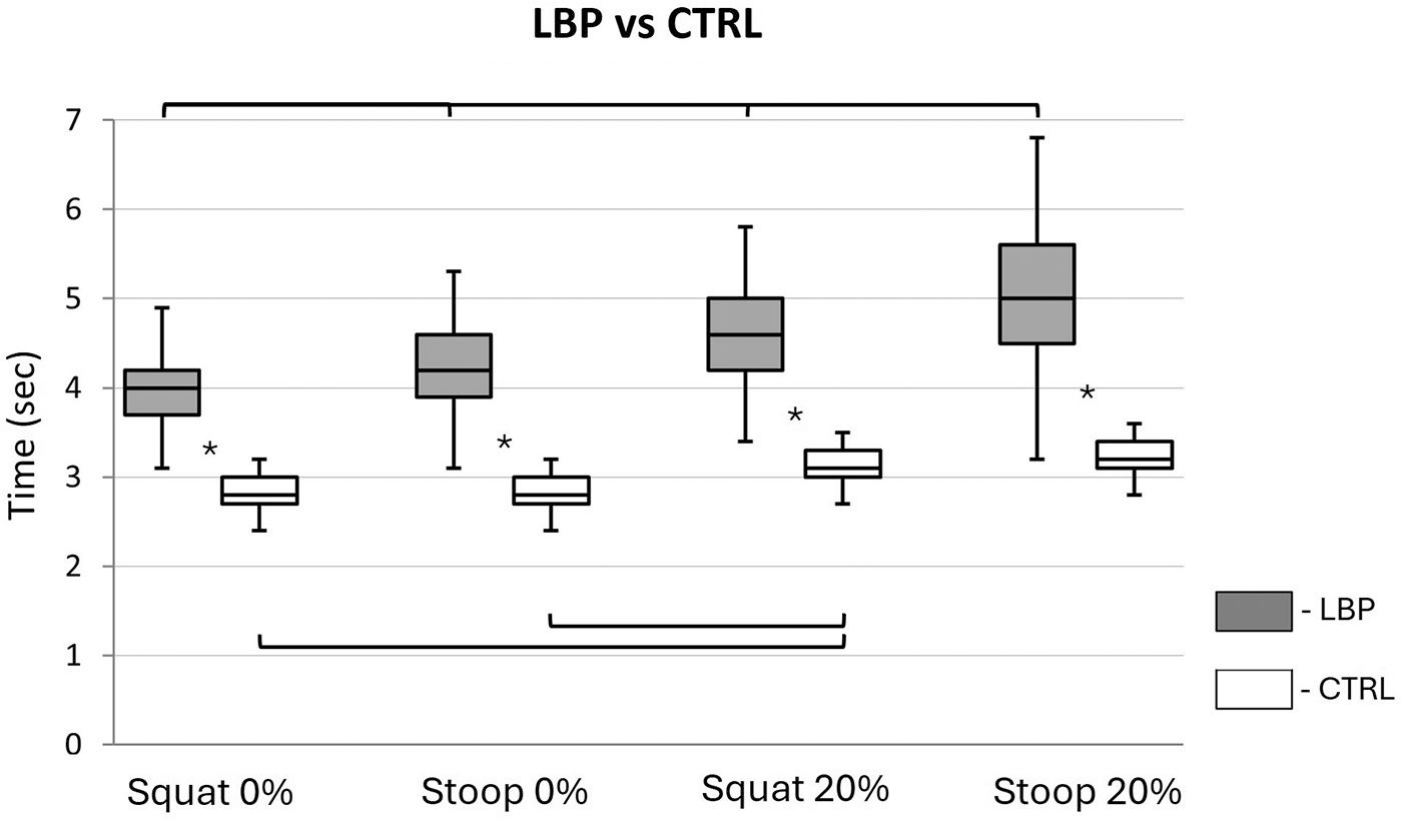
Lifting tasks with four conditions. The box represents the mean and 95% CI, and whiskers show the SD. Brackets indicate significant within-group differences (*p* < 0.004); *significant between-group differences (*p* < 0.001).

Pairwise comparison of LBP patients and asymptomatic controls shows significant group differences, with lower values for the control group in every condition (*p* < 0.001; Table 2, Figure 2).

**Table 2:** Functional lifting test with four conditions [mean ± SD (95% CI), time (s)]: LBP = 48, control=37.

Condition	LBP	Control	*p*-Value^a^
Squat0%	4.0 ± 0.9^a^ (3.7, 4.2)	2.8 ± 0.4 (2.7, 3.0)	<0.001
Stoop0%	4.2 ± 1.1^a^ (3.9, 4.6)	2.8 ± 0.4^a^ (2.7, 3.0)	<0.001
Squat20%	4.6 ± 1.2^a^ (4.2, 5.0)	3.1 ± 0.4 (3.0, 3.3)	<0.001
Stoop20%	5.0 ± 1.8^a^ (4.5, 5.6)	3.2 ± 0.4 (3.1, 3.4)	<0.001

a Not normally distributed; Mann–Whitney *U*-test (*B*-corrected *α*=0.0125).

ANOVA revealed a statistically significant interaction effect [Greenhouse–Geisser *F*(1.738, 144.247) = 5.885, *p* = 0.005]. Furthermore, there were significant main effects for condition [Greenhouse–Geisser *F*(1.738, 144.247) = 35.414, *p* < 0.001] and group [*F*(1, 83) = 52.252, *p* < 0.001]. *Post-hoc* analysis showed statistically significant differences between all four conditions in the LBP group (*p* < 0.001) and between squat0% and squat20% (*p* < 0.001) and stoop0% and squat20%(*p* = 0.004) in the control group.

There was no association between pain intensity in LBP patients and the TTC in any of the four conditions. Pearson's *r* ranged from 0.008 to 0.129 (*p* > 0.05).

### Test–retest reliability

**Figure 3:**
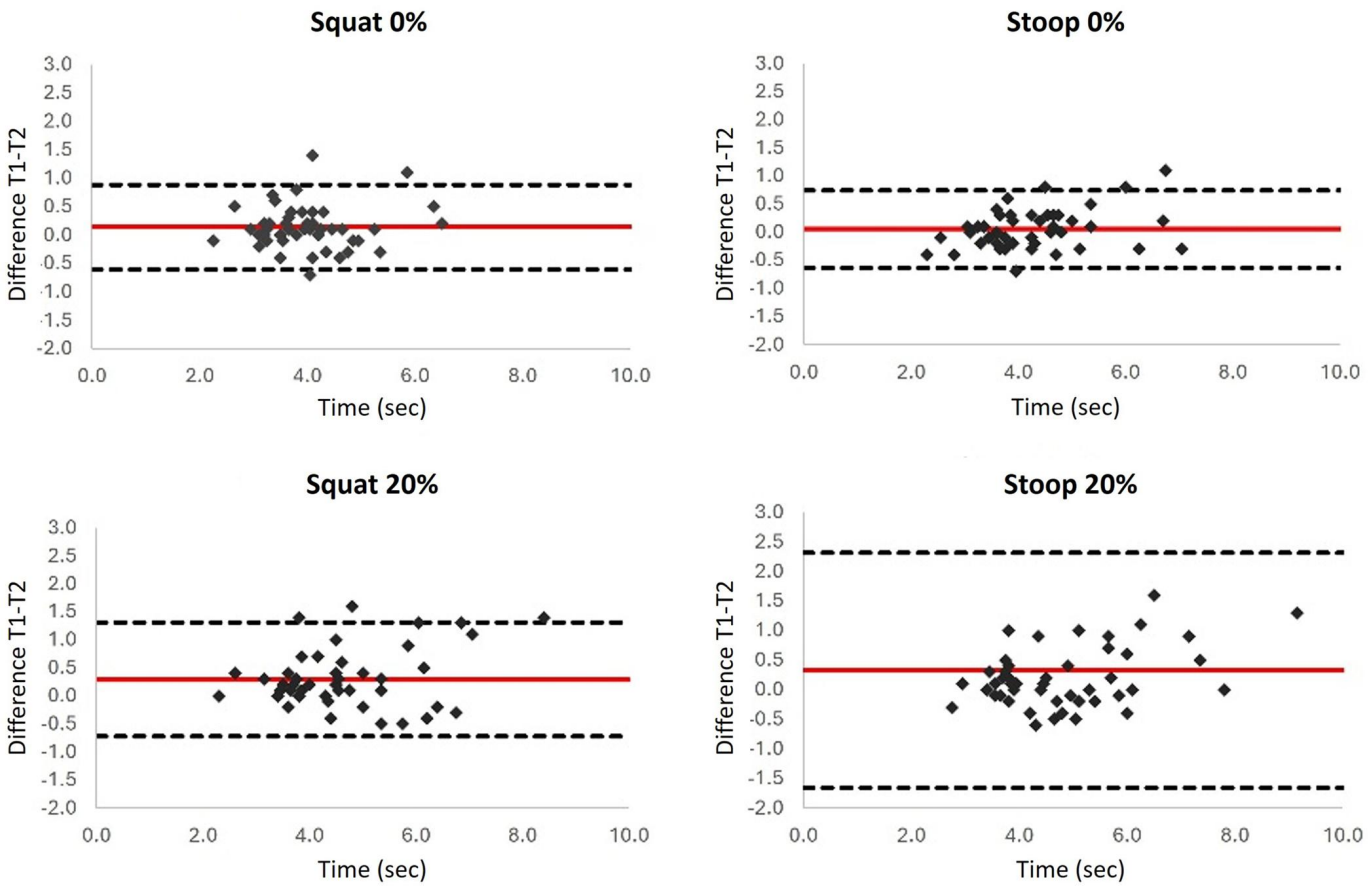
Bland–Altman plots for the LBP group (*n* = 48).

**Figure 4:**
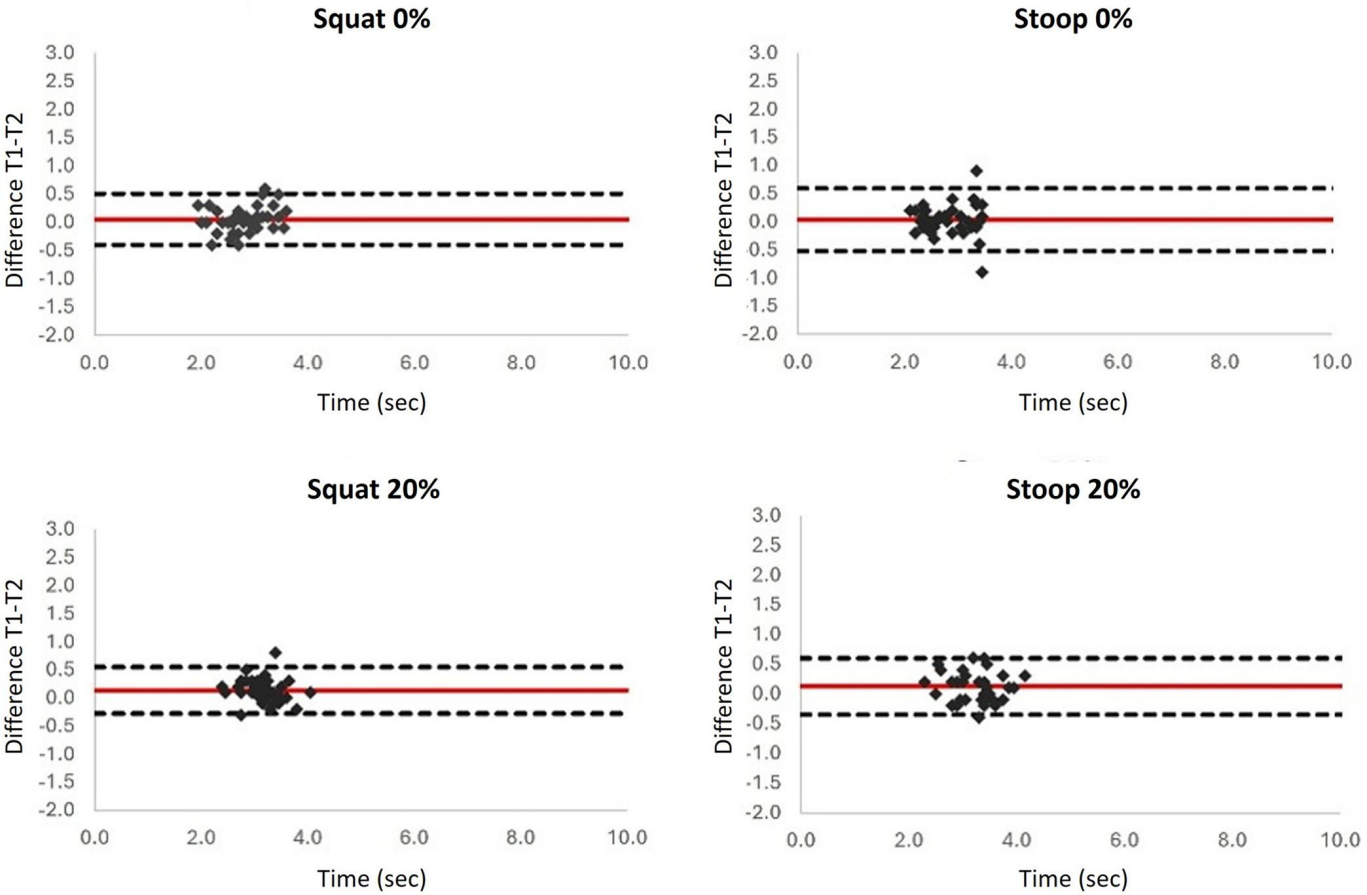
Bland–Altman plots for the control group (*n* = 37).

Results for test–retest reliability are presented in Tables 3, 4 and Figures 3, 4. In both groups, ICC ranged from 0.85 to 0.95, TRV ranged from 6% to 8%, systematic error (bias) ranged from 0.05 to 0.34 s, SEM ranged from 0.14 to 0.72 s, and SDC ranged from 0.27 to 1.41 s.

**Table 3:** Time (s) as mean ± SD for four lifting conditions and reliability measures for the LBP group (*n* = 48).

Condition	T1	T2	ICC 2.1 (95% CI)	TRV ± SD %	Bias ± LoA	SEM, SDC
Squat0%	4.1 ± 0.9	4.0 ± 0.9	0.90 (0.82–0.95)	7.1 ± 7.0	0.14 ± 0.75	0.27, 0.53
Stoop0%	4.3 ± 1.1	4.2 ± 1.0	0.95 (0.91–0.97)	6.4 ± 4.9	0.05 ± 0.69	0.25, 0.49
Squat20%	4.7 ± 1.3	4.4 ± 1.2	0.89 (0.72–0.95)	8.4 ± 8.2	0.29 ± 1.02	0.36, 0.71
Stoop20%	5.2 ± 2.2	4.9 ± 1.5	0.85 (0.74–0.91)	8.4 ± 8.5	0.32 ± 1.99	0.72, 1.41

**Table 4:** Time (s) as mean ± SD for four lifting conditions and reliability measures for the control group (*n* = 37).

Condition	T1	T2	ICC 2.1 (95% CI)	TRV ± SD %	Bias ± LoA	SEM, SDC
Squat0%	2.8 ± 0.5	2.8 ± 0.4	0.87 (0.76–0.93)	6.2 ± 5.8	0.05 ± 0.45	0.16, 0.31
Stoop0%	2.9 ± 0.4	2.8 ± 0.4	0.80 (0.65–0.89)	6.8 ± 6.2	0.04 ± 0.56	0.20, 0.39
Squat20%	3.2 ± 0.4	3.1 ± 0.4	0.81 (0.52–0.91)	6.2 ± 5.1	0.14 ± 0.41	0.14, 0.27
Stoop20%	3.3 ± 0.4	3.2 ± 0.5	0.83 (0.64–0.92)	7.0 ± 5.2	0.12 ± 0.48	0.17, 0.33

### ROC analysis

**Figure 5:**
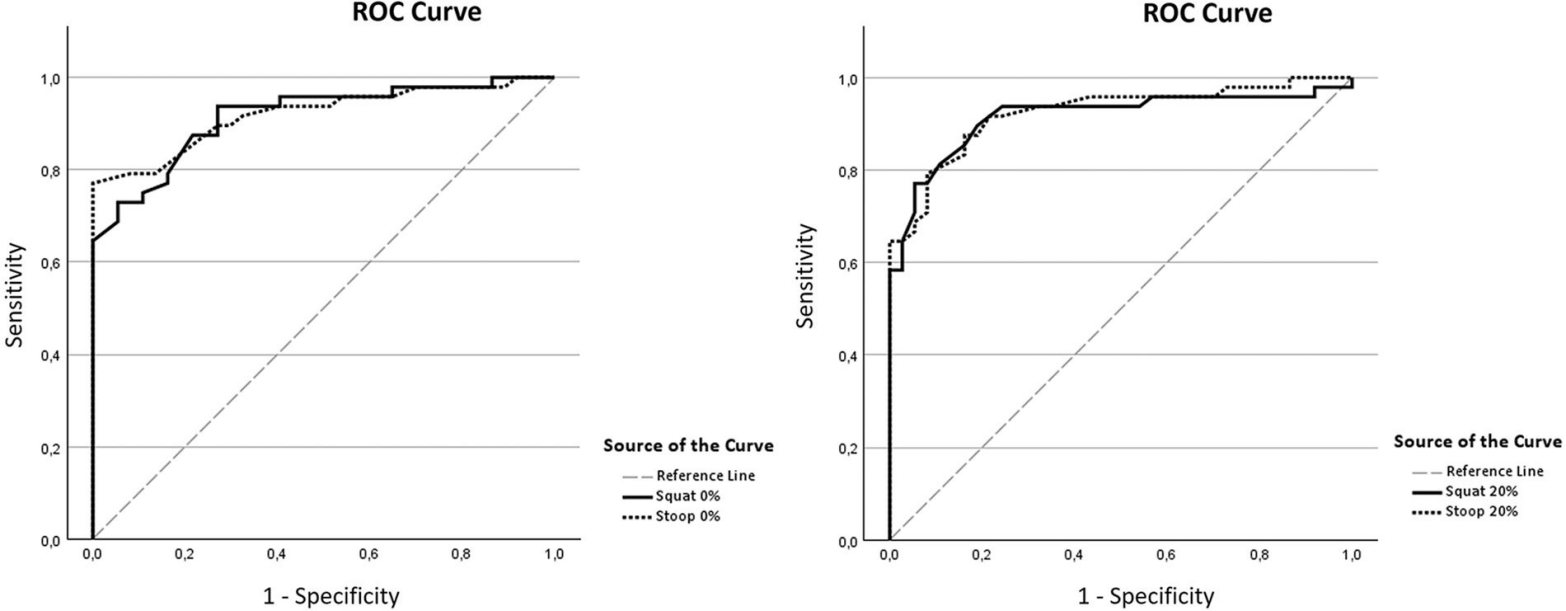
ROC curve for unloaded (left) and loaded (right) conditions.

ROC analysis results are shown in Table 5 and Figure 5. AUC showed excellent results, ranging from 0.915 to 0.923 across all test conditions. The cut-off value ranged from 3.5 to 3.7 s.

**Table 5:** ROC analysis with cutoff values (s) based on Youden's *J* calculation differentiating LBP and control groups.

Condition	AUC (95% CI)	Model quality	*p*-Value	Youden's *J*	Cutoff value (s)
Squat0%	0.916 (0.857–0.974)	0.86	<0.001	0.675	3.475
Stoop0%	0.919 (0.860–0.978)	0.86	<0.001	0.771	3.525
Squat20%	0.915 (0.850–0.979)	0.85	<0.001	0.717	3.650
Stoop20%	0.923 (0.865–0.980)	0.87	<0.001	0.713	3.625

## Discussion

The study evaluated test–retest reliability and discriminatory power of a standardized functional lifting assessment for detecting functional impairment in LBP patients compared with asymptomatic controls. High test–retest reliability across all four lifting conditions, with a test–retest variability of 6%–8%, together with a clear discrimination between individuals with and without LBP (AUC > 0.91), indicate the applicability of the test in research and clinical practice.

Functional lifting assessment presents a usual everyday task comparable with established clinical tests, such as the “sit-to-stand” or “timed up and go” test (23), but is more focused on LBP-specific functional impairment regarding alterations during spinal flexion and extension movements (4, 23). The results of the study show that movement velocity during different lifting conditions is significantly impacted by back pain. Although there is no association between time to completion and pain intensity, LBP patients move more slowly and with greater variability in TTC, especially during load lifting. A previous systematic review also reported that LBP patients commonly bend more slowly and more stiffly, with less lumbar range of motion, and use more bent knees while lifting compared with asymptomatic subjects (4, 23). It has been discussed that these altered lifting techniques are most likely a consequence of wanting to protect the spine from high loads, a result of pain-avoidance behavior, or a result of adherence to the commonly communicated more safe squat lifting technique (4). Consistent with this belief, eight participants declined to participate in at least one lifting task due to fear of pain. While there is evidence that higher movement velocity is associated with higher spinal loads (14), *in vivo* studies have not proven that squat lifting with “straight back” rather than a stoop manner, as commonly educated by health care practitioners and physical therapists, is protective and results in lower spinal loading (4). This is also reflected by the similar results in TTC for squat and stoop lifting in the present investigation.

Previous studies have investigated the reliability, validity, and responsiveness of different lifting tests in the LBP population (20, 24). The “progressive isoinertial lifting task,” which consists of freestyle lifting of a box with a standardized weight four times within 20 s, with the weight increased in each new cycle and the number of completed lifting stages documented, was also found to be reliable (ICC 0.92) but not clinically useful (25) and was not responsive to changes over time (26). A modified progressive isoinertial lifting task applied by Strand et al. (27), using maximum weight lifted rather than the number of completed lifting cycles as the outcome measure, showed similarly high reliability (ICC 0.91, SEM 3.1 kg, SDC 8.4 kg); however, the generalizability of these results is limited, as they are based on nine chronic LBP patients not responding to a rehabilitation intervention. They applied a second lifting test consisting of repeatedly lifting a weighted box (4 kg for women, 5 kg for men) from the ground onto a table and back down, counting the number of lifts within 1 min, and the test only showed moderate reliability in a preliminary study (ICC 0.71, SEM 3.2, and SDC 9.0; unpublished data) (27). Magnussen et al. (21) examined the reliability and validity of a back performance scale, including one freestyle lifting test consisting of lifting a box with a 5 kg sandbag from the floor to a table and back repeatedly during 1 min, with the observer scoring on an ordinal scale from 0 for “no activity limitation” to 4 for “major activity limitation.” They found high inter-rater reliability in scoring test performance at the same time and only moderate test–retest reliability (kappa=0.55). Validity was not reported for single tests. Although these studies report moderate to high reliability and mostly acceptable validity in different lifting tests, information on responsiveness to changes over time remains limited (20). A major limitation of these tests is the use of standardized weights that differentiate only between men and women, without accounting for inter-individual constitutional differences that result in different relative loading. Furthermore, freestyle lifting does not allow monitoring of transitions in lifting technique from squat-like to stoop-like manor, which may be related to changes in pain and functional impairment (15). Therefore, the present study developed a lifting test focusing on movement velocity, as this has been found to be significantly reduced in individuals suffering from LBP (4). It is an inexpensive, time-effective, and easy-to-administer functional assessment that can be applied in research and clinical practice. It only requires basic standardization with a marker on the floor, a short explanation of the knee position, one familiarization trial for each task, and a simple normalization calculation to apply the proper relative load. Furthermore, the test differentiates between squat and stoop box-lifting techniques, without and with additional weight, allowing testing across tasks ranging from a less provoking to a potentially more provoking task and from a frequently proclaimed “safe” to an “unsafe” lifting technique (15). As some participants refused to perform some or all tasks due to fear of pain, not only the time required for execution but also mere participation in each task are outcome measures of interest and potential indicators of changes in functional impairment. As fear-avoidance beliefs can affect task participation and task performance [e.g., movement velocity (6)], future studies may consider incorporating assessments of psychological factors, such as the fear-avoidance beliefs questionnaire (28), to investigate their potential association with TTC.

The presented lifting test clearly discriminates between asymptomatic individuals and LBP patients. Overall, the control group completed all lifting tasks more quickly and with less variation than LBP patients. ROC analysis indicated a threshold of 3.5 s for 0% squat and stoop lifting and 3.6–3.7 s for 20% squat and stoop lifting to differentiate between the two groups. These results are in line with findings from previous studies reporting that LBP patients move more slowly and stiffer during spinal movement, bending, and lifting tasks (4, 29). The lack of correlation between movement velocity and pain intensity and the relatively greater variability in time to completion in the LBP group may be explained by the fact that functional (dis)ability is not directly tied to pain perception. Previous studies have discussed that fear of pain and movement avoidance play a significant role in deconditioning and impairment of daily activities (30). Therefore, addressing functional limitations by including exercise interventions in the treatment regimen can improve performance even with LBP and can be beneficial for pain perception (30).

## Limitations

The study presents some limitations. The sequence of lifting conditions was standardized for all participants, progressing from lifting without additional weight to lifting with additional weight from squat (widely considered “safer”) to stoop (commonly considered a more “unsafe” condition) to increase compliance in participation. A standardized resting period of 15 s between single-test trials was used to avoid or mitigate cumulative fatigue. However, it cannot be ruled out that the within-group differences between “unloaded” and loaded conditions and the differences between loaded squat and stoop in LBP patients are to some degree affected by this sequence of testing and fear of pain due to frequent lifting, comparable to the limitation reported by von Arx (14). Furthermore, the simple and easy-to-administer manual measurement of TTC using a stopwatch is susceptible to human reaction-time error. Therefore, providing and adhering to clear and standardized start and stop criteria is essential to ensure adequate clinical reproducibility. Moreover, test–retest reliability was based on consecutive task execution at a single measurement time point, omitting information on potential day-to-day variability in the investigated outcome of TTC. Finally, what remains to be investigated is the responsiveness of this lifting assessment in detecting changes in functional performance and pain. This could be achieved by applying the outcome measure of this lifting assessment, along with established functional tests and validated questionnaires addressing pain and functional limitations in LBP patients in controlled exercise intervention studies. Furthermore, future studies should clarify whether the assessment may serve as a screening tool in the therapeutic setting to help define the stage of training initiation in LBP patients.

## Conclusion

The findings of the current investigation show that the functional lifting test is reliable across all four conditions, performed by LBP patients and controls, with a test–retest variability of 6%–8%. Overall, asymptomatic participants complete all lifting tasks more quickly and with less variation in time to completion compared to LBP patients. The test can clearly discriminate between people with and without LBP (AUC > 0.91). It is quickly performed, easy to administer, simple in standardization, and provides a useful assessment of the presence of functional limitations in LBP patients in the clinical and therapeutic setting.

## Data Availability

The raw data supporting the conclusions of this article will be made available by the authors, without undue reservation.
